# Efficient Tumor Eradication at Ultralow Drug Concentration via Externally Controlled and Boosted Metallic Iron Magnetoplasmonic Nanocapsules

**DOI:** 10.1021/acsnano.2c05733

**Published:** 2022-12-05

**Authors:** Arnon Fluksman, Aritz Lafuente, Zhi Li, Jordi Sort, Silvia Lope-Piedrafita, Maria José Esplandiu, Josep Nogues, Alejandro G. Roca, Ofra Benny, Borja Sepulveda

**Affiliations:** † Institute for Drug Research (IDR), School of Pharmacy, Faculty of Medicine, The Hebrew University of Jerusalem, 9190501Jerusalem, Israel; ‡ Catalan Institute of Nanoscience and Nanotechnology (ICN2), CSIC and BIST, Campus UAB, 08193 Bellaterra, Barcelona, Spain; § Universitat Autònoma de Barcelona, Campus UAB, 08193 Cerdanyola del Vallès, Barcelona, Spain; ∥ ICREA, Pg. Lluís Companys 23, 08010Barcelona, Spain; ⊥ Instituto de Microelectronica de Barcelona (IMB-CNM, CSIC), Campus UAB, 08193 Bellaterra, Barcelona, Spain

**Keywords:** nanocapsules, photothermal therapy, magnetic manipulation, paclitaxel, breast cancer

## Abstract

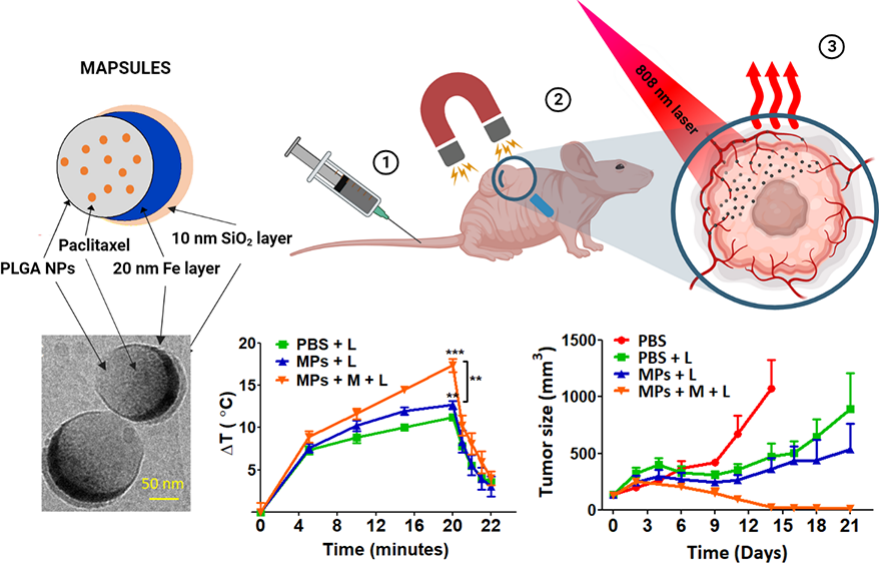

With the aim to locally enhance the efficacy of cancer nanotherapies, here we present metal iron based magnetoplasmonic drug-loaded nanocapsules (MAPSULES), merging powerful external magnetic concentration in the tumor and efficient photothermal actuation to locally boost the drug therapeutic action at ultralow drug concentrations. The MAPSULES are composed of paclitaxel-loaded polylactic-*co*-glycolic acid (PLGA) nanoparticles partially coated by a nanodome shape iron/silica semishell. The iron semishell has been designed to present a ferromagnetic vortex for incorporating a large quantity of ferromagnetic material while maintaining high colloidal stability. The large iron semishell provides very strong magnetic manipulation via magnetophoretic forces, enabling over 10-fold higher trapping efficiency in microfluidic channels than typical superparamagnetic iron oxide nanoparticles. Moreover, the iron semishell exhibits highly damped plasmonic behavior, yielding intense broadband absorbance in the near-infrared biological windows and photothermal efficiency similar to the best plasmonic nanoheaters. The *in vivo* therapeutic assays in a mouse xenograft tumor model show a high amplification of the therapeutic effects by combining magnetic concentration and photothermal actuation in the tumor, leading to a complete eradication of the tumors at ultralow nanoparticle and drug concentration (equivalent to only 1 mg/kg PLGA nanoparticles containing 8 μg/kg of paclitaxel, i.e., 100–500-fold lower than the therapeutic window of the free and PLGA encapsulated drug and 13–3000-fold lower than current nanotherapies combining paclitaxel and light actuation). These results highlight the strength of this externally controlled and amplified therapeutic approach, which could be applied to locally boost a wide variety of drugs for different diseases.

Nanotherapies based on organic nanoparticles, e.g., liposomes, dendrimers, micelles, polymeric particles, conjugated or encapsulated with drugs have emerged as a valuable tool for overcoming the side effects of chemotherapies by increasing both the circulation time and accumulation of the drug inside solid tumors by exploiting their high drug loading capacity and the currently debated enhanced permeability and retention (EPR) in the tumor vasculature.^1,2^ However, nanotherapies have not yet met these expectations due to the final low nanoparticle concentration in solid tumors.^3,4^ A recent analysis of nanotherapies in murine models showed that only an average 0.7% of the injected nanoparticles arrive at the tumor.^5^ These hurdles are behind the clinical approval of only very few nanotherapies to date, which have not demonstrated the high-efficacy gains expected from the preclinical assays.^6^ Thus, strategies enhancing the delivery and efficacy of the therapeutic agents could reduce the time course of treatments while reducing treatment frequency and dosages.

Magnetic nanostructures have been proposed to externally control and improve nanotherapy delivery.^7−10^ However, these nanostructures are mostly based on small superparamagnetic iron oxide nanoparticles, which limit the drug loading capacity and exhibit a weak magnetic moment that severely hampers their efficient external actuation with magnetic fields. The use of this type of particle stems from the difficulty to achieve colloidally stable ferromagnetic nanoparticles by chemical synthesis, as they tend to irreversibly aggregate by their intense magnetic dipolar interactions in the absence of a magnetic field.^11^ Interestingly, multifunctional dimeric nanoparticles combining plasmonic and magnetic moieties have also been proposed for simultaneous light and magnetic actuation.^8,9^ Nevertheless, these systems generally show a similar weak magnetic response and limited drug loading capabilities.^10,12−14^ Therefore, the development of drug vehicles with improved capacity of external control is required, as a way to achieve more efficient local release of the drug and to enhance the therapeutic activity.^15^

Here we present a biodegradable metal iron based magnetoplasmonic nanocapsule, which combines a large drug-loading capacity with the following unprecedented set of multiactuation and therapeutic capabilities: (i) highly efficient magnetic manipulation and concentration via magnetophoretic forces keeping a high colloidal stability, (ii) intense magnetic resonance imaging and strong optical absorption to noninvasively visualize and track the nanocapsules, and (iii) highly efficient and broadband optical heating in the two biological windows as adjuvant therapy. This set of features has enabled magnetic concentration of the nanocapsules at the tumor region and eradication of the tumors in mice at very low nanocapsule and drug concentrations by exploiting the external photothermal actuation.

## Results and Discussion

### Fabrication and Physicochemical Properties

**Figure 1:**
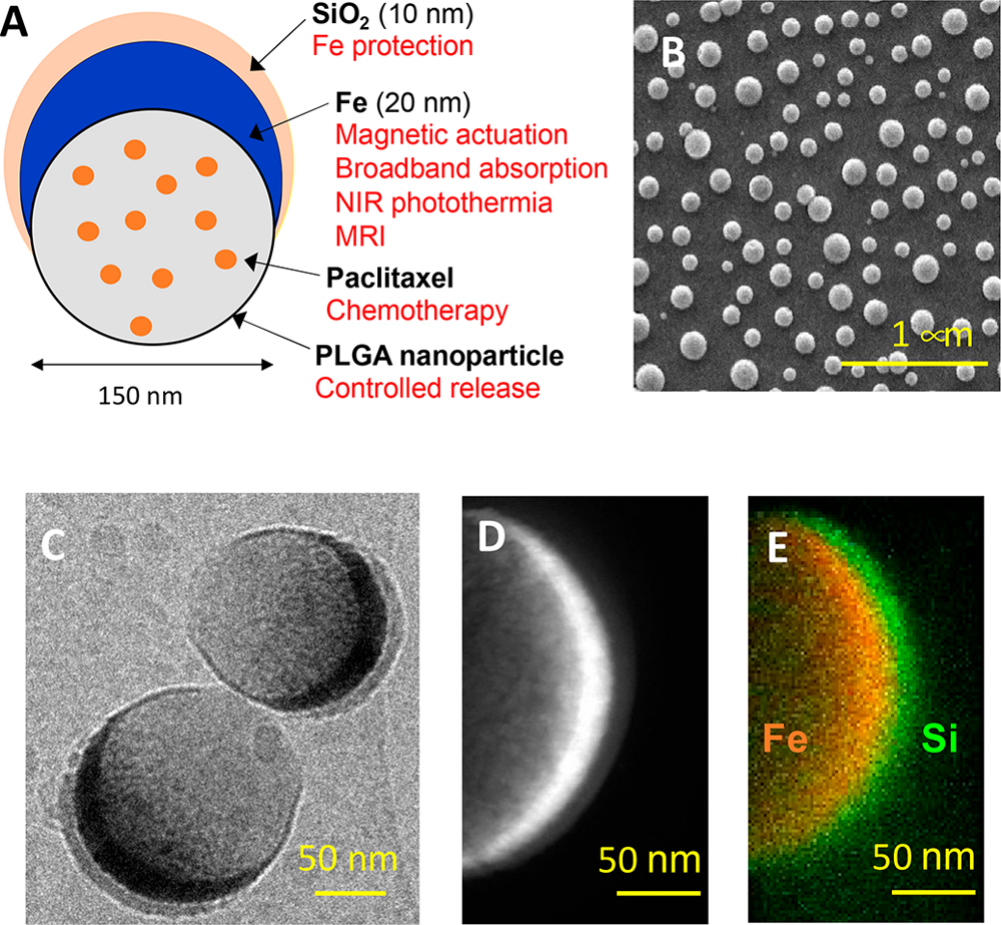
**MAPSULES structural properties**. (A) Schematic of the drug-loaded ferromagnetic nanocapsules components and their functionalities. (B) SEM image of the self-assembled PLGA nanoparticles capped with Fe (20 nm) and SiO_2_ (10 nm). (C) TEM images of the ferromagnetic nanocapsules after dispersion in water for 3 h. (D) High-magnification TEM image of the semishell to show the thickness of the Fe and SiO_2_ layers, and (E) EDX mapping at the energies corresponding to Fe and Si atoms.

The active biodegradable magnetoplasmonic nanocapsules (MAPSULES) are composed of acid terminated polylactic-*co*-glycolic-acid (PLGA) nanoparticles (diameter *ca*. 150 nm) loaded with the anticancer drug paclitaxel, which are partially covered by metallic Fe (20 nm) and a SiO_2_ (10 nm) layers (Figure 1A).

The MAPSULES fabrication is based on a cost-effective and scalable combination of bottom-up and top-down processes, involving nanoparticle synthesis, self-assembly, and physical vapor deposition.^16,17^ In this case, the fabrication process can be divided into three steps (see Methods): (i) synthesis of the paclitaxel-loaded PLGA nanoparticles by the emulsification-evaporation method, showing an average hydrodynamic diameter of 150 nm (polydispersity index 0.07 ± 0.01), a zeta potential of −30 mV given by the carboxylic groups at the particle surface (Figure S1E), and paclitaxel loading efficiency of 38%, which corresponds to a drug payload of 0.8% (i.e., 0.8 mg paclitaxel/100 mg PLGA × 100); (ii) electrostatic self-assembly of the loaded PLGA nanoparticles on a positively charged solid support (e.g., silicon wafers or Kapton films) by exploiting the negative charge of the PLGA particles, which generates a uniform monolayer of randomly distributed and well separated PLGA nanoparticles (Figure 1B), and (iii) deposition of the Fe (20 nm) and SiO_2_ (10 nm) layers by electron beam evaporation to form the “nanodome” structure (Figures 1C–E and S1A,B). The metallic Fe layer provides the strong ferromagnetic and highly damped plasmonic properties, while the SiO_2_ protects the Fe from a rapid oxidation and provides negative charges to improve the colloidal stability.

The MAPSULES are finally dispersed in water or buffer by mild ultrasonication. The obtained polydispersity index of the dispersed MAPSULES by dynamic light scattering was 0.2. However, we consider that this value is overestimated due to the inherent anisotropy of the MAPSULES, which show different scattering cross sections depending on their orientation with respect to the polarization of the incident light, as will be discussed below. Once dispersed in water, the PLGA hydrolysis triggers the nanocapsule degradation and the paclitaxel release. According to the high-performance liquid chromatography (HPLC) measurements, approximately 40% of the drug is released within the first 4 days (Figure S1F). This release profile correlates with the known burst kinetic effect in PLGA nanoparticles (NPs).^18,19^ The full degradation of the PLGA nanoparticle nearly takes one month, as can be observed in Figure S1G.

The diameter of the nanocapsules and the thickness of the Fe layer have been designed to simultaneously achieve high saturation magnetization, vortex magnetic structure, and intense broadband optical absorbance covering the whole near-infrared range.

**Figure 2:**
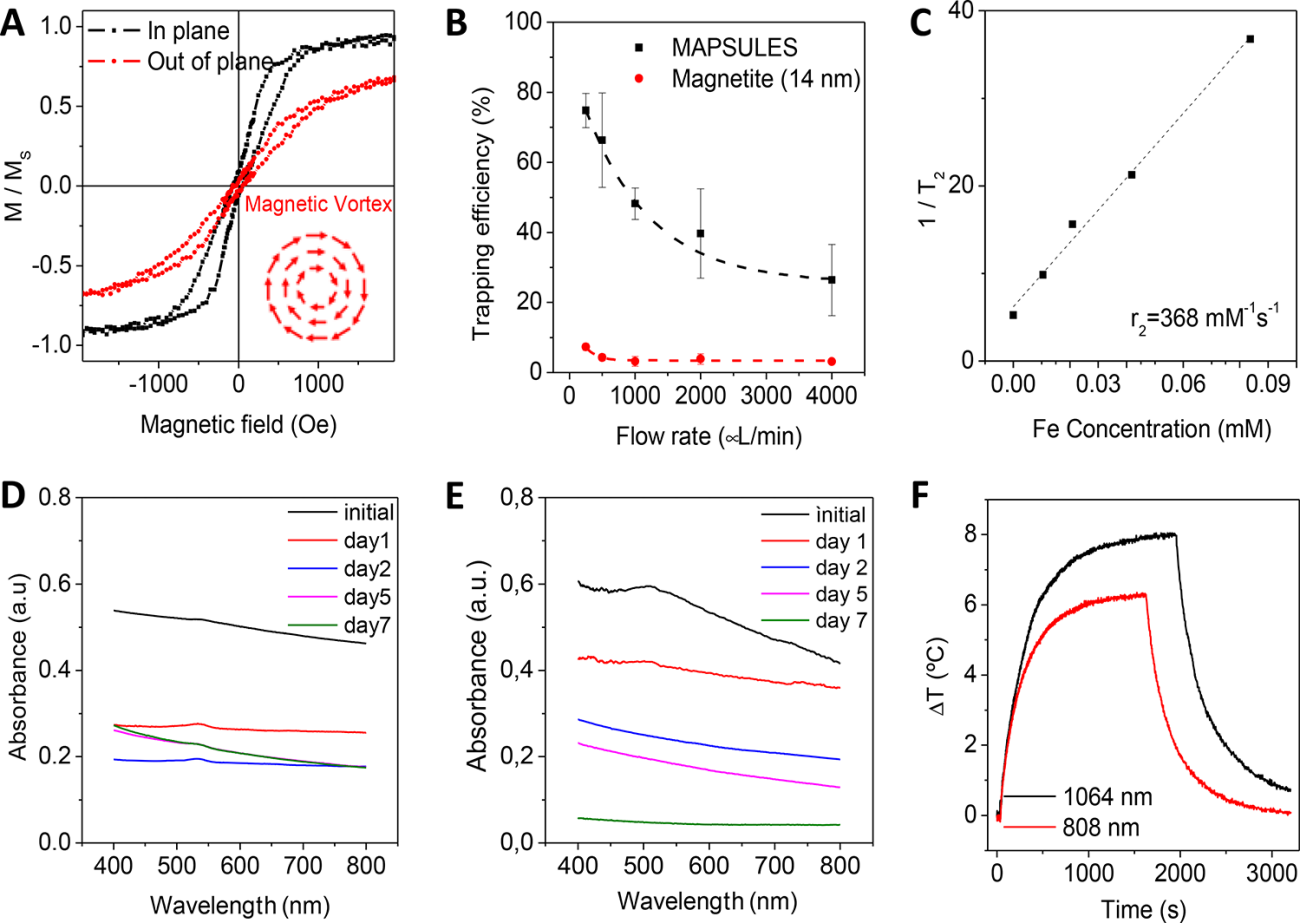
**MAPSULES magnetic and optical properties**. (A) In-plane and out-of-plane normalized hysteresis loops showing the preferential in-plane ferromagnetic vortex structure (inset image shows a schematic of the magnetic vortex configuration). (B) Magnetic trapping efficiency in a microfluidic channel at increasing flow rates (see experimental set up in Figure S2). (C) Nuclear magnetic resonance *T*_2_ contrast and *r*_2_ relaxivity. (D,E) Evolution of the nanoparticle absorbance during degradation in PBS at 37 °C at (D) pH 7 or (E) pH 4. (F) Demonstration of the efficient optical heating in the first and second biological windows, using 808 and 1064 nm lasers, respectively (incident light power 200 mW, beam diameter 5.2 mm – 0.95 W/cm^2^, MAPSULES concentration 9.75 μg/mL, and volume 600 μL).

The MAPSULES show in-plane magnetization with a vortex magnetic configuration (Figure 2A) with a near zero remanence and a high susceptibility, i.e., *M*(*H*) slope.^20^ As a consequence, the magnetic moment and the dipolar interactions between the nanocapsules are negligible in the absence of magnetic field, which is crucial to keep stable colloidal dispersions. Importantly, the metallic iron layer offers a much higher saturation magnetization (*M*_*S*_ = 218 emu/g) than that of typical superparamagnetic iron oxide nanoparticles, such as Fe_3_O_4_ (*M_S_* = 92 emu/g). The larger magnetization and the high colloidal stability of the metallic iron semishell enable fabricating nanoparticles with more powerful magnetic features. As a comparison, a MAPSULE with a diameter of 150 nm exhibits a saturation magnetic moment *ca*. 300-fold larger than that in superparamagnetic 16 nm edge magnetite nanocubes. Over that size, the magnetite cubes become ferromagnetic, and their colloidal stability is markedly reduced.^21^ As the magnetophoretic forces are proportional to the magnetic moment, the high values that can be achieved with the MAPSULES are the key for their efficient magnetic trapping and accumulation using moderate external magnetic field gradients. To demonstrate the magnetic actuation capability via magnetophoretic forces, we compare the magnetic trapping efficacy of the MAPSULES and 14 nm edge magnetite nanocubes by a spherical FeNdB magnet (diameter 18.8 mm, surface magnetic field 7.1 kOe, and field gradient according to Figure S2B) in a microfluidic channel mimicking the flow conditions in blood vessels (see Methods and Figure S2). The percentage of trapped nanoparticles by the magnet is quantified by measuring the relative decrease of the absorbance (Δ*A*/*A*) of the nanocapsule dispersions that flow through the microfluidic channel. As can be observed in Figure 2B, even in a single fluidic pass, an extremely high trapping efficiency (>50%) can be achieved for flow rates lower than 1 mL/min, which are in the range of the blood flow in mice (i.e., from 90 μL/min in the spleen to 1800 μL/min in the liver).^22^ For higher speeds, the trapping percentage is stabilized at *ca*. 20% per pass. Importantly, the trapping efficiency of the MAPSULES is over 10-fold higher than that of the superparamagnetic magnetite nanocubes. It is worth highlighting that this large enhancement is achieved despite the large PLGA nanoparticle acting as nonmagnetic cargo in the MAPSULES and the much higher viscous friction in the liquid imposed by their much larger hydrodynamic diameter. These results highlight their potential for efficient magnetic trapping at the blood capillaries surrounding the tumor area.

The high magnetization of the metal iron layer also offers noninvasive and high-contrast imaging of the MAPSULES by nuclear magnetic resonance (NMR). The MAPSULES exhibit a very strong *T*_2_ signal in NMR showing a relaxivity *r*_2_ of 368 s^–1^ mM^–1^ (Figure 2C), which is much higher than that of commercial contrast agents based on iron oxides (e.g., Feridex and Resovist; *r*_2_ ≈ 120–189 s^–1^ mM^–1^).^23^

Regarding the optical properties (Figure 2D), the MAPSULES present an intense broadband absorbance that slightly decreases toward shorter wavelengths due to the scattering reduction in this region. The undefined broad absorption band is a result of the highly damped plasmonic behavior of the metallic iron, whose imaginary part of the dielectric constant is much higher than that in the typical plasmonic metals, such as Ag and Au.^24,25^

Interestingly, the absorbance variations can be used to analyze the degradation of the iron semishell during time. As can be observed in Figure 2D, the absorbance spectrum of the nanocapsules shows a fast decrease in the whole visible range during the first 4 days (*ca*. 46% decrease) when the capsules are kept at 37 °C and pH 7, which is accompanied by an increase of the absorbance in the ultraviolet region of the spectrum. This optical evolution is consistent with the partial oxidation of the metallic iron layer, which initially shows plasmonic enhanced absorption in the near-infrared spectral region, into iron oxides and hydroxides showing increased absorption in the blue and ultraviolet regions.^26^ In addition, the absorbance of the supernatant in the ultraviolet region followed a continuous increase that is related to the release of partially water-soluble PLGA moieties and Fe^3+^ ions to the media (Figure S3). A negligible spectral contribution from the surfactants and paclitaxel is expected in the supernatant considering the fabrication process, which eliminates any remaining surfactants used in the synthesis of the PLGA nanoparticles and the low solubility and small quantity of the paclitaxel compared to the PLGA (i.e., 0.8%). In addition, as the nanoparticle internalization inside cells generally leads to nanoparticle trapping inside lysosomes with low internal pH (i.e., between 4 and 5), we have also analyzed the MAPSULES degradation at pH 4. As can observed in Figure 2E, the degradation at lower pH is substantially faster, with an almost complete loss of the absorbance after 7 days. The rapid degradation is due to the destabilization of the SiO_2_ layer at low pH, thereby exposing the Fe layer, whose corrosion is also enhanced by the higher solubility of the iron ions at low pH. This behavior is very relevant to promote a fast biodegradation of the MAPSULES but enables the preservation of the optical and magnetic properties during the necessary time (i.e., a few hours post injection) to externally control and enhance the therapeutic effects, as it will be shown below.

The highly damped plasmonic behavior of the metallic iron semishell induces a large reduction of the scattering cross section of the nanocapsules while maintaining a high and broadband absorption cross section (Figure S4A–C). This is due to the higher penetration of the light electromagnetic field inside the iron film compared to the penetration in typical plasmonic metals.^24^ The nanodome shape is also very relevant from the photothermal perspective, as it offers several benefits with respect to other typical shapes of similar dimensions, such as iron nanodiscs or nanospheres. The simulations in Figure S4C show that the absorption cross section, which is the responsible for the optical heating, is approximately equal for the MAPSULES and iron nanospheres despite the amount of iron is 5-fold lower for the MAPSULES. Moreover, the MAPSULES exhibit a flatter absorption spectrum, showing similar absorption in the first and second biological windows. Compared to iron nanodiscs, the absorption cross section is substantially higher for the MAPSULES, and it is more isotropic, as the absorption variations for the different orientations of the nanostructure with respect to the light polarization are much weaker.

These optical features are ideal for achieving high photothermal conversion efficiencies. Indeed, the photothermal conversion efficiency in the first (wavelength 808 nm) and second biological windows (wavelength 1064 nm) are 63 and 67%, respectively (Figure 2F), which is comparable with state-of-the-art nanoplasmonic heaters.^27,28^ The slightly increased temperature variation at 1064 nm observed in Figure 2F is a consequence of the water heating contribution at this wavelength (Figure S4E).

### *In Vitro* Analysis

**Figure 3:**
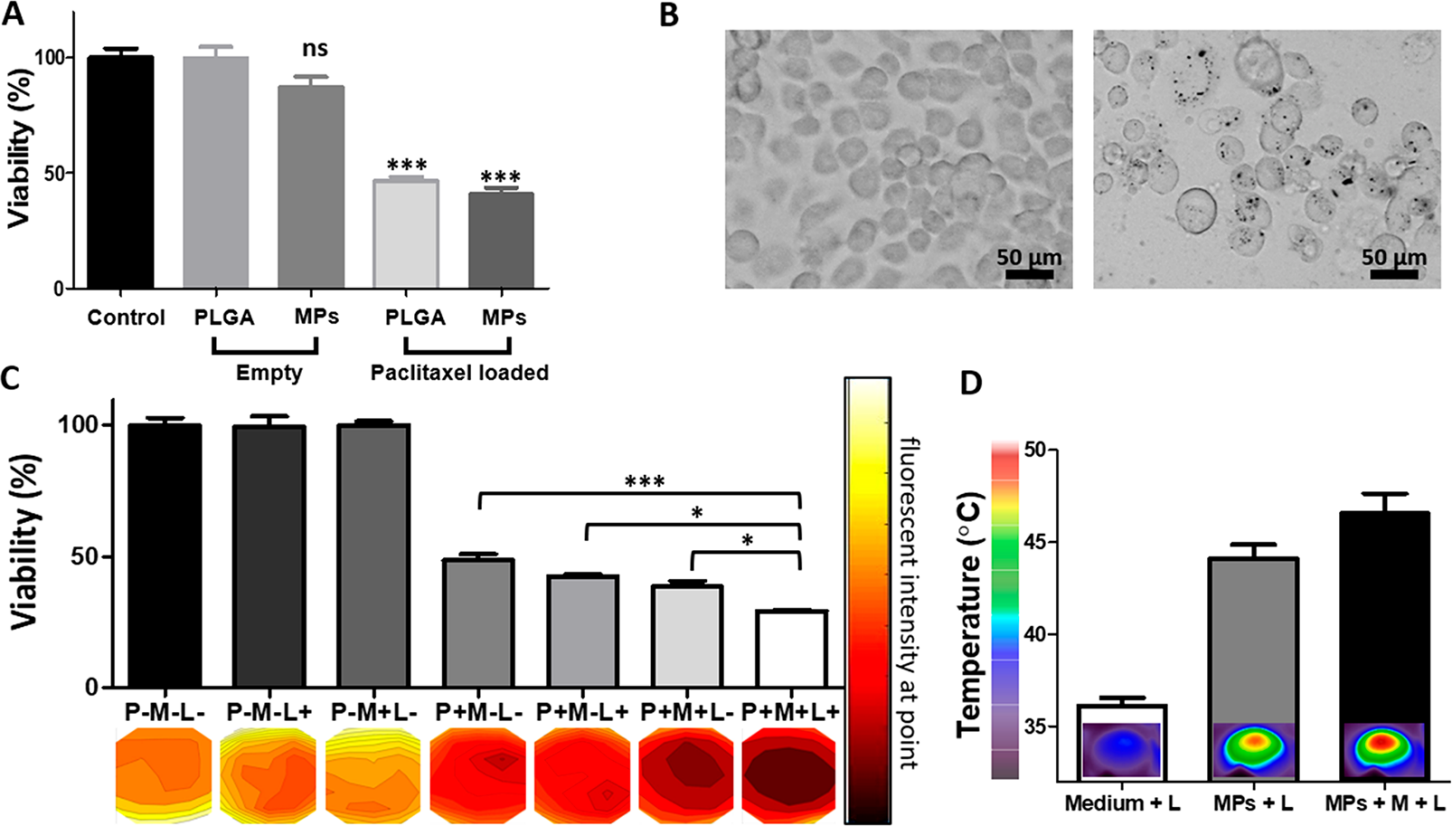
***In vitro* characterization**. (A) Comparison of the viability of the unloaded and paclitaxel-loaded PLGA nanoparticles and the MAPSULES, keeping identical nanoparticles and drug concentration (10 μg/mL of PLGA nanoparticles and 0.08 μg/mL of paclitaxel). (B) Bright field images of the untreated (left) and MAPSULES-treated (right) MDA-MB-231 cells. The internalized MAPSULES are observed as black dots inside the cells. (C) Comparison of the viability under the different treatment conditions with the MAPSULES (P+), laser irradiation (L+), and magnetic concentration (M+). (D) Analysis of the photothermal effects in the cultured MDA-MB-231 cells treated with the MAPSULES and the MAPSULES after magnetic concentration compared to the untreated cells. * *p* < 0.05, *** *p* < 0.001

To assess the biomedical potential of the MAPSULES, we first analyzed their toxicity and therapeutic activity *in vitro* by their exposure to cultures of human breast adenocarcinoma MDA-MB-231 cells under different treatment conditions (Figure 3).

We first compared the effects of the loaded and unloaded PLGA nanoparticles and the MAPSULES. The tumor cells were incubated for 48 h with equal PLGA and paclitaxel concentrations of 10 μg/mL of PLGA and 0.08 μg/mL of paclitaxel. In the case of the MAPSULES, this value corresponds to a mass concentration of 28 μg/mL due to the mass contribution of the Fe and SiO_2_ layers. The unloaded PLGA nanoparticles and the MAPSULES without drug did not reduce the viability of the cells compared to the untreated control cells (Figure 3A). In contrast, for both the paclitaxel-loaded PLGA nanoparticles and the MAPSULES, the viability was reduced to 46 and 40%, respectively, due to the effect of the paclitaxel release (Figure 3A).

These results confirmed the low toxicity of the MAPSULES, the preservation of the paclitaxel release, and its therapeutic activity even in the MAPSULES morphology, indicating that the fabrication process does not degrade their properties. Moreover, the bright field images of the MAPSULES-treated MDA-MB-231 cells, taken after 4 h of incubation, confirm the internalization of the MAPSULES, as shown by black dots inside the cells (Figure 3B).

Next, we analyzed the effects of the magnetic concentration and photothermal actuation of the MAPSULES on the viability of the cancer cells (Figure 3C,D). We compared the passive administration of the MAPSULES with the administration under the effect of a magnetic field gradient generated by a spherical FeNdB magnet (diameter 12.7 mm, surface magnetic field 7.1 kOe) placed under the center of the well for 30 min and/or the effect of the local photothermal actuation with a near-infrared laser in the first biological window (wavelength 808 nm, beam diameter 11 mm, power density 0.95 W/cm^2^) during 10 min. In all the cases, the MAPSULES concentration was equal to the previous experiment of Figure 3A. The viability was analyzed 24 h after treatment using a MTT assay as well as with fluorescence areal scanning with calcein AM staining for viability distribution evaluation in each well. The results showed a complete lack of effect of the magnetic field and light irradiation in the absence of nanoparticles (P-M-L+ and P-M+L-) and a similar viability decrease to Figure 3A in the passively administrated MAPSULES (P+M-L-), and this effect was homogeneous across the well, as shown by the fluorescence intensity map of Figure 3C. The viability was slightly reduced in the case of the light-treated samples (P+M-L+) with passive administration, in which the temperature increased up to 44 °C (Figure 3D). In the case of the magnetically actuated MAPSULES (P+M+L- and P+M+L+), the viability reduction was higher, the therapeutic effect was more localized at the center of the well due to the magnetic concentration, and the laser irradiation could increase this effect, as a consequence of the temperature increase up to 47 °C (Figure 3D). Note that the light-induced amplification of the therapeutic effects could be substantially increased by irradiating the samples inside an incubator at 37 °C. Moreover, notice that the obtained temperature variation cannot be compared to that of Figure 2F, as the experimental conditions in terms of particle distribution, buffer volume, illuminated area, and light path length in the sample are very different.

### *In Vivo* Toxicity and Biodistribution Analysis

**Figure 4:**
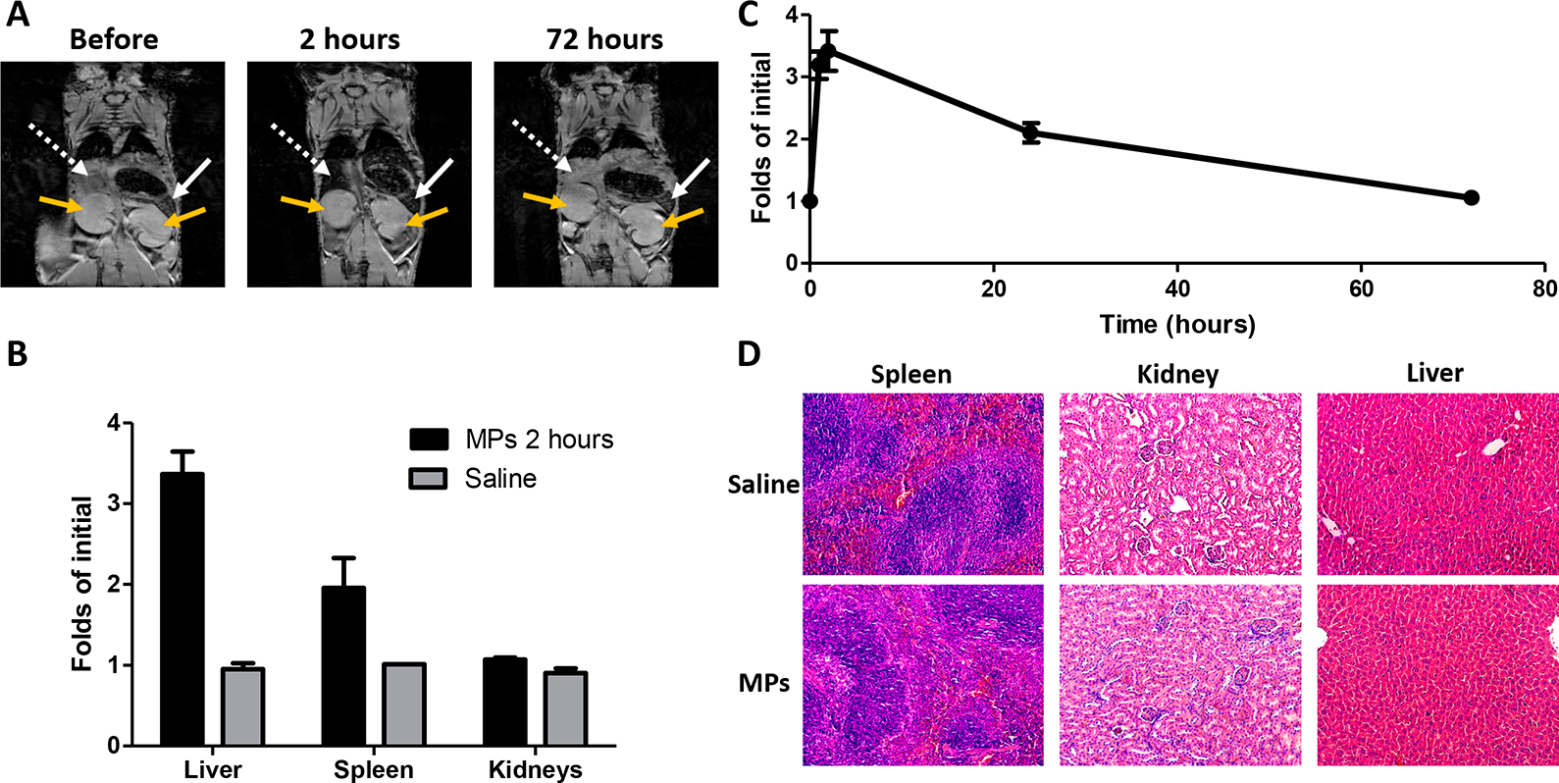
***In vivo* biodistribution analysis**. (A) Magnetic resonance *T*_2_-weighted images of hsd/foxn1 nude mice before and after tail vein injection of 2.5 mg/kg of unloaded MAPSULES (white dashed line points to the liver, the white line points to the the spleen, and the yellow line points to the kidneys). (B) Comparison of the organs’ accumulation at 2 h after injection with respect to the saline-treated mice. (C) Liver biodistribution over time. (D) Hematoxylin and eosin stained sections of the spleen, kidney, and liver of mice 72 h post intravenous injection of saline or unloaded MAPSULES.

Once the low cytotoxicity and the therapeutic activity were demonstrated *in vitro*, we analyzed *in vivo* the MAPSULES biodistribution and toxicity in Hsd:Athymic Nude-Foxn1^nu^ mice by exploiting their intense *T*_2_ contrast in NMR. We injected 2.5 mg/kg (i.e., 100 μg) of unloaded MAPSULES in mice (*N* = 2) via tail vein injection, and we monitored their distribution by *T*_2_-weighted imaging at different times (Figure 4A).

The NMR images showed a very clear decrease in the T_2_ signal in the liver and spleen 2 h after the injection, while there was not a significant signal decrease in other organs, such as kidneys or muscle (Figure 4A,B). The *T*_2_ signal in the liver already increased at 24 h postinjection, and it was almost fully recovered after 72 h (Figure 4C), indicating that the MAPSULES were cleared by the organism. To confirm the lack of toxicity, the spleen, kidneys, and liver were harvested 72 h postinjection and stained by hematoxylin and eosin (H&E) for histological analysis to assess the MAPSULES effects in these organs, compared to saline injection (Figure 4D). Despite the initial accumulation of the MAPSULES in these organs shown by NMR, there was no tissue damage at 72 h. Moreover, the mice did not suffer any weight loss or any noticeable toxicity effects during the whole 3 week assay (Figure S7), thus indicating that the MAPSULES are safe. These results suggest a secretion pathway through the liver, as expected for the size and surface charge of the MAPSULES and the passive delivery.^29−32^ It should be noted that in contrast to other studies focused on nonbiodegradable Au particles,^33^ whose secretion rate is rather slow, a faster clearance of the MAPSULES due to their biodegradability is expected. To corroborate this hypothesis, we carried out mass spectrometry analysis of the liver and kidneys. As Figure S5A shows, the iron levels in the liver increased right after the injection but quickly decreased after 24 h, which correlates with the data acquired by NMR and histopathology. On the other hand, Si increasingly accumulated in the liver for 72 h, but no damage was observed in the organ. In contrast, neither the iron nor the silicon levels raised in the kidneys, which confirms that the elimination route was not renal.

### *In Vivo* MAPSULES Treatment

#### Short-Term Effects

**Figure 5:**
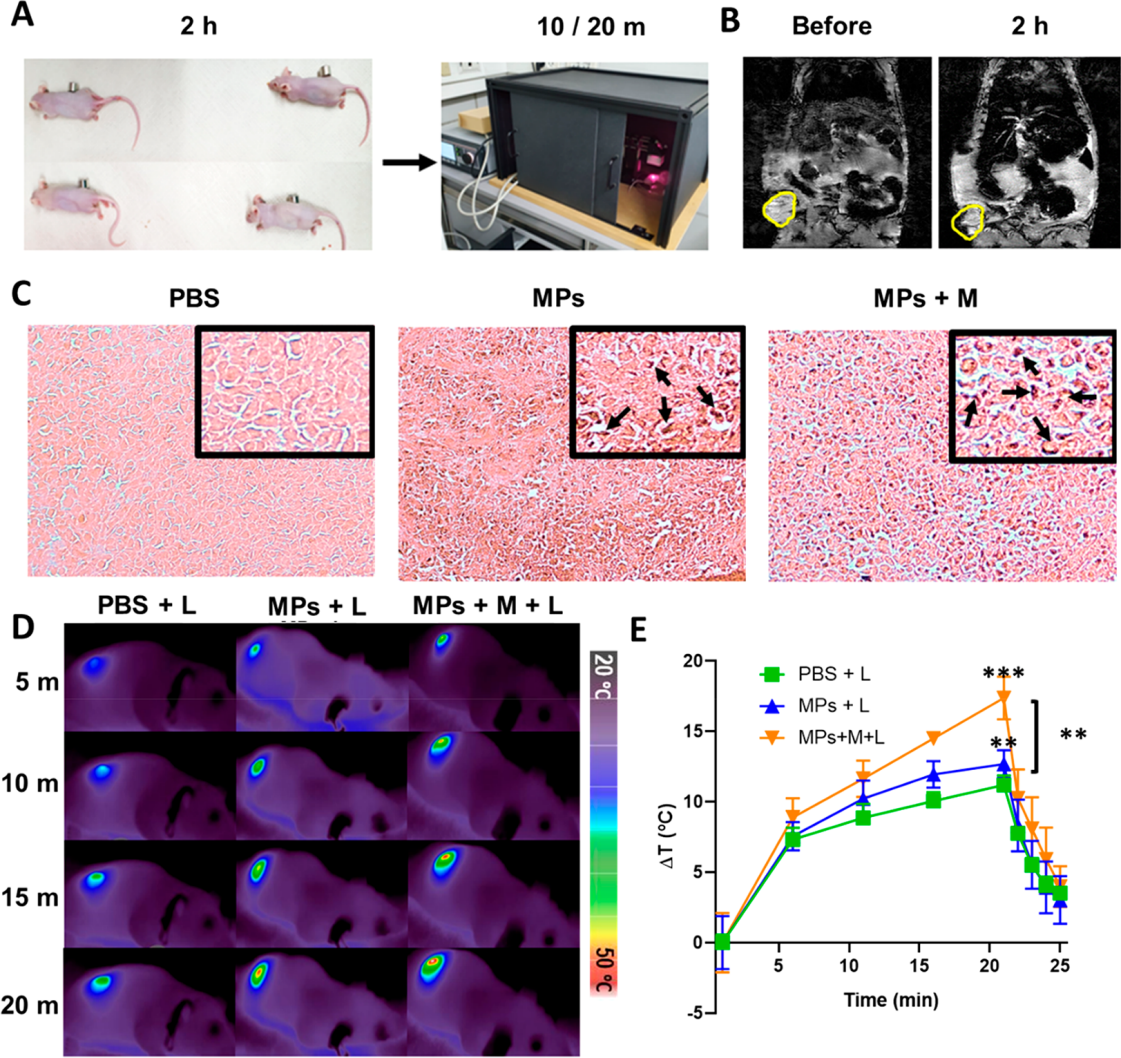
***In vivo* short-term effects**. (A) *In vivo* setup for the MAPSULES magnetic and light actuation. (B) NMR images of tumor bearing mice before and 2 h post MAPSULES injection and magnetic concentration (tumor circled in yellow). (C) Histological examination for iron detection using Prussian blue staining. MAPSULES accumulation regions are indicated by the black arrows in the zoom insets. (D) Representative thermal images of the laser-treated mice after intravenous injection of PBS or MAPSULES with and without magnetic concentration. (E) Plot of the average temperature increases as a function of time for each treatment condition.

To investigate the potential of the paclitaxel-loaded MAPSULES for magnetic and optically enhanced cancer chemotherapy, we first compared the treatment response in Hsd:Athymic Nude-Foxn1^nu^ female MDA-MB-231 tumor bearing mice under six different conditions: (i) PBS control (PBS), (ii) PBS and laser actuation (PBS+L), (iii) MAPSULES (MPs), (iv) MAPSULES and magnetic concentration (MPs+M), (v) MAPSULES and laser actuation (MPs+L), and (vi) MAPSULES and magnetic concentration plus laser actuation (MPs+M+L). The mice were placed on a 33 °C warming pillow and anesthetized for 2.5 h during the treatment sequence of tail intravenous injection, magnetic concentration, and light actuation. A volume of 150 μL (PBS or MAPSULES) was administered via tail vein injection. In the case of the MAPSULES treatment, the total mass concentration was 2.8 mg/kg of MAPSULES, which corresponds to 1 mg/kg of paclitaxel-loaded PLGA nanoparticles and only 8 μg/kg of paclitaxel. These concentrations are substantially lower than the typical free and PLGA loaded with paclitaxel in mice, which are in the 1.0–5.0 mg/kg range (Table 1).^34−38^ The magnetically actuated groups were exposed to a cylindrical FeNdB magnet (diameter 12 mm, height 10 mm, surface magnetic field 5 kOe, and field gradient according to Figure S2C) placed next to the tumor for 2 h right after the MAPSULES injection (Figure 5A). The laser actuated groups were irradiated for 20 min by the 808 nm laser with a power density of 0.95 W/cm^2^ (beam diameter 11 mm), and the temperature of the mice was monitored by a thermal camera (Figure 5A).

**Table 1:** Comparison of PLGA and Paclitaxel (PCX) Concentrations of PLGA and Paclitaxel (PCX) in *In Vivo* Treatments

	PLGA [mg kg^–1^]	PCX [mg kg^–1^]	Reference
MAPSULES	0.8	8.0 × 10^–3^	This work
PLGA-PEG NP	142.8	1.0	[^34^]
Aptamer-PLGA	47.6	1.0	[^35^]
Ab-PLGA	–	2.0	[^36^]
MNP/T7-PLGA	14.8	4.0	[^37^]
PLGA-PEG NC	833	5.0	[^38^]

Interestingly, 2 h after the injection, the NMR images of the tumor bearing mice exposed to the magnet revealed a clear decrease in the *T*_2_ signal at the tumor site, demonstrating the ability to magnetically accumulate the MAPSULES in the tumor area (see the marked yellow circle in Figure 5B).

To determine if the MAPSULES could penetrate inside the tumor mass, the tumors were harvested 2 h after the PBS or MAPSULES injection with or without magnetic concentration, sectioned, and stained using Prussian blue/eosin to detect the presence of iron. As shown in Figure 5C, while only few iron signatures corresponding to the MAPSULES were observed in the tumor after the passive intravenous injection, high levels of iron could be detected inside the tumor after the magnetic concentration (indicated by the black arrows in Figure 5C). This result confirms that the magnetic strength of the MAPSULES enables a highly efficient enrichment, not only next to the skin, where the magnetic field gradient is higher, but also inside the tumor. To quantify the amount of MAPSULES in the tumor, we carried out mass spectrometry analysis of the tumors harvested 24 h post injection. Figure S5B demonstrates that the magnetic concentration clearly increased the amount of iron and, consequently, the MAPSULES concentration inside the tumor. Considering the mass of the tumors (*ca.* 200 mg) and the quantity of injected particles, it can be estimated that 0.9% of the MAPSULES were trapped inside the tumor by passive delivery and 2.2% by magnetic concentration.

Next, we analyzed the photothermal effects induced by the near-infrared laser exposure for the different treatment conditions. As can be observed in Figure 5D,E, the group with passively delivered MAPSULES (MPs+L) exhibited only *ca*. 2 °C higher temperature increase induced by the laser compared to the control (PBS+L). In contrast, for magnetically concentrated treatments, a significant 8 °C temperature increase enhancement was achieved after 20 min of illumination. This substantially higher temperature increase confirmed the capacity to magnetically amplify the MAPSULES concentration in the tumor. Note that the 2.4-fold higher MAPSULES concentration in the tumor with magnetic actuation determined by mass spectrometry was obtained 24 h after the intravenous injection. However, a substantially higher MAPSULES concentration in the tumor is expected right after the magnetic concentration (i.e., 2 h post injection). Such a concentration increase can be estimated from the temperature rise in the tumor. As can be observed in Figure S5C, the photoinduced temperature rise is proportional to the particle concentration, at least up to 20 μg/mL of iron. In the case of the *in vivo* experiments, the temperature increase in the passive delivery was only 2 °C higher than the control, whereas the magnetically actuated mice achieved 8 °C enhancement with respect to the control, thereby suggesting a 4-fold higher concentration of the MAPSULES 2 h post injection. The difference between the concentrations obtained by mass spectrometry (24 h post injection) and photothermal effects (2 h post injection) is probably due to the MAPSULES that were weakly attached at the periphery of the tumors, which were cleared through the tumor blood vessels after removal of the magnet. These results suggest that a longer magnetic actuation time could be used to keep the 4-fold MAPSULES concentration increase in the tumor, which could enhance even more the therapeutic effect of the released paclitaxel.

The observed temperature increases in the control group (*ca*. 10 °C) are due to the non-negligible absorption of the 808 nm laser light by the skin, blood, and tumor tissues.

#### Long-Term Therapeutic Effects

**Figure 6:**
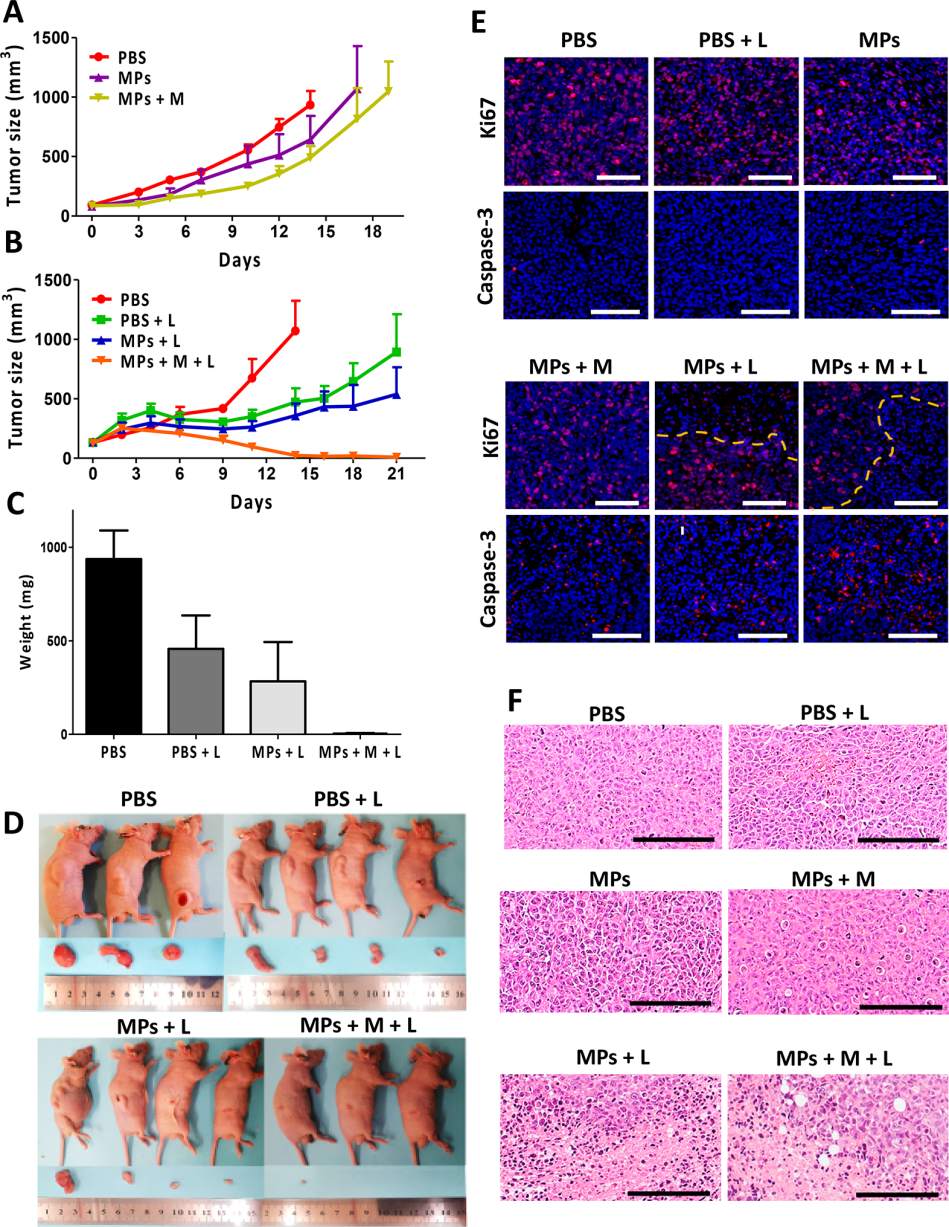
***In vivo* magnetic concentration and laser radiation effects**. (A) Tumor growth curves in mice groups for 21 days or until reaching the volume of the exclusion criterion after magnet exposure treatments. (B) Tumor growth curves in mice groups after different laser radiation treatments for 21 days or until reaching the volume of the exclusion criterion after the different laser radiation treatments. (C) Tumor average weight for the different treatment conditions at the experiment end point given by the exclusion volume (PBS) or 21 days. (D) Representative images of the treated mice and harvested tumors at the laser radiation experiment end point. (E) Fluorescent images of stained sections for Ki67 (pink) (top row) and cleaved caspase-3 (red) (bottom row) and counterstained with DAPI for cell nucleus imaging (blue). (F) H&E staining of tumor sections harvested at the end point (scale bar 200 μm).

To evaluate the efficiency and long-term effects of the MAPSULES under the different treatment conditions, the treated mice were monitored until reaching the exclusion criterion of over 1000 mm^3^ of tumor volume or 21 days post injections as experiment end points. First, we examined the effect of magnetically driven accumulation of the MAPSULES in tumor growth (Figure 6A). The PBS injected group (PBS) reached the exclusion volume (>12-fold from initial size) at day 14 post injection. In contrast, the mice with passively injected MAPSULES and the MAPSULES with magnetic concentration (MPs and MPs+M) showed a substantial decrease in the tumor growth compared to the control, and the exclusion volume was reached at days 17 and 19, respectively (Figure 6A). Although the magnetic accumulation of the MAPSULES in the tumor was not sufficient for the complete tumor eradication, the tumor growth reduction is noteworthy taking into account the very low concentration of the injected nanoparticles and drug compared to other therapies based on PLGA encapsulated paclitaxel (Table 1).^34−38^

Considering these promising therapeutic results, we next evaluated the effect of laser radiation in the tumor area on the MAPSULES therapeutic activity, using the same MAPSULES concentration and laser irradiation conditions described above. The analysis of the tumor growth over time (Figure 6B–D) showed an enhanced therapeutic effect for the passively delivered MAPSULES (MPs+L) irradiated for 20 min compared to the irradiated control group (PBS+L) and the nonirradiated groups at the 21 day end point, showing tumor volumes of 550 and 900 mm^3^, respectively. Strikingly, complete eradication of the tumors in the group with magnetic concentration and light actuation (MPs+M+L) was already observed at day 14, which corresponded to the volume exclusion end point for the PBS control. This large amplification effect by light radiation suggests that the laser power or irradiation time could have been reduced to achieve similar enhanced therapeutic activity and complete elimination of the tumor with the magnetically concentrated MAPSULES under these experimental conditions. Such reduction could also minimize the observed skin burn effect at the irradiated regions in this experiment, although the mice completely recovered from this superficial side effect at the experimental end point (Figure S8). The observed reduction in the tumor growth with laser irradiation in the absence of MAPSULES is consistent with previous results of the effect of near-infrared light heating on breast cancer tumor bearing mice, which showed that the locally induced optical hyperthermia could induce immune cell activation and infiltration within the tumor,^39^ being these effects highly dependent on the final temperature. To gain insights into the effects of light irradiation without MAPSULES injection, we carried out an *in vivo* experiment in mice irradiated with similar intensity but during shorter illumination times (see Figure S6). The results clearly showed a negligible decrease of the tumor growth rate with respect to the control at shorter illumination times. These results again suggest that an irradiation time of 15 min would have probably been the ideal condition to maximize the photothermal effects of the MAPSULES with respect to the PBS+L condition.

To examine the cellular effects of the treatments, the tumors were harvested, and representative tumors from each group were analyzed by H&E, Ki67, and cleaved capase-3 staining (Figure 6E,F). The H&E staining revealed the presence of apoptotic bodies in the magnetically actuated groups, MPs+M and MPs+M+L, while more severe effects with additional visible coagulative necrosis were observed in the light-treated groups, MPs+L and MPs+M+L. Conversely, no noticeable damage was observed in the other treated groups (Figure 6F). The enhanced apoptosis in the magnetically treated mice is related to the enhanced paclitaxel concentration inside the tumors due to the magnetic MAPSULES enrichment. In contrast, the observed necrosis can be associated with the enhanced photothermal effect induced by the MAPSULES. To evaluate the aggressiveness of the tumors, the sectioned tumors were stained for the detection of the proliferation marker Ki67 (Figure 6E). Strong fluorescent signal was detected in all the samples; however, light actuated groups, MPs+L and MPs+M+L, exhibited a noticeable clear region where tissue was less proliferative and cell density was reduced (Figure 6E, top row). On the other hand, cleaved caspase-3 staining was performed to assess apoptotic cell death, showing elevated signal in the MPs+M, MPs+L, and MPs+M+L groups (Figure 6E). These results are consistent with the H&E staining, confirming that the magnetic concentration enables increased paclitaxel levels in the tumor, whereas the local photothermal actuation drastically amplifies its effects. These findings confirm the synergistic effect of chemo-photothermal combined therapy, where the whole is larger than the sum of its parts.^40^ It is worth highlighting the achieved results using only one ultralow drug dose by intravenous injection, which enabled the tumor ablation by combining the magnetic concentration and the photothermally boosted chemotherapeutic effects, especially if they are compared to other light and paclitaxel multimodal therapies, which required multiple injections and/or a much higher drug amount, i.e., between 13- and 3000-fold higher than the MAPSULES treatment (Table 2).^41−47^

**Table 2:** Comparison of the MAPSULES Conditions and Other Multimodal Therapies Combining Paclitaxel and Light Actuation

	Optical settings		Total PCX [mg kg^–1^]	*In vivo* conditions			Ref
	Type	Conditions		Tumor	Injection	Growth inhibition	
MAPSULES	PTT (808 nm)	0.95 W cm^–2^	8.0 × 10^–3^	MDA-MB-231	iv	Ablation^c^	This work
Poly(Ru/PTX)^a^	PDT	671 nm, 0.2 W cm^–2^	15.0	4T1	iv	65%	[^41^]
Shape changeable NPs^a^		650 nm, 0.2 W cm^–2^	0.09	4T1	iv	85%	[^42^]
Liposomes^a^		450 nm, 0.2 W cm^–2^	24.0	PC3	it	Ablation^c^	[^43^]
Chitosan@Au NPs	PTT (808 nm)	1.2 W cm^–2^	0.7	MDA-MB-231	iv	97%	[^44^]
CuS NPs^a^		1 W cm^–2^	0.16	4T1	iv	Ablation^c^	[^45^]
Gelatin-PtNPs^b^		—	20.0	4T1	iv	83%	[^46^]
Polymeric micelle^b^		0.07 W cm^–2^	15.0	4T1	iv	Ablation	[^47^]

a Several complete treatments (light + injection).

b Several injections.

c Not all the tumors from the group were ablated (iv: intravenous, it: intratumoral, PTT: photothermal therapy, PDT: photodynamic therapy).

## Conclusions

In conclusion, the presented MAPSULES constitute a cutting-edge nanotherapeutic concept capable of merging externally controlled therapies with noninvasive imaging and drastically enhanced therapeutic effects at very low drug concentrations. The high magnetic strength and colloidal stability of the metal iron semishells in the MAPSULES are the key features for enabling efficient external magnetic actuation via magnetophoretic forces. The strong magnetism also provides an outstanding *T*_2_ contrast in NMR to noninvasively visualize the MAPSULES biodistribution and magnetic accumulation in the tumor. On the other hand, the high optical absorbance with minimal scattering provided by the highly damped plasmonic behavior of the Fe semishell enables highly efficient optical heating in the first and second NIR biological windows. The therapeutic activity of the MAPSULES *in vitro* has demonstrated the capacity to preserve the activity of the paclitaxel during the MAPSULES fabrication process. The MAPSULES have shown *in vivo* a total absence of side effects and the expected biodistribution with accumulation in the liver after passive intravenous injection. The magnetically actuated mice have shown a notable increase of the MAPSULES retention in the tumor, as demonstrated by the NMR images, the mass spectrometry measurements, the enhanced photothermal effects, and the histopathological analysis of the harvested tumors. The obtained value of delivery efficiency inside the tumor by passive injection (0.9%) is within the expected range in the literature, showing an average of 0.6% for the organic particles and 0.8% for the inorganic particles.^3^ In contrast, the magnetic concentration provides a powerful strategy to substantially increase the particle dose in the tumor (2.2% at 24 h post injection). Notably, the magnetic actuation was limited to only 2 h due to ethical considerations related to the mice wellbeing, despite the small size of the employed magnet (*ca.* 1 cm^3^). A longer magnetic actuation would possibly retain a larger fraction of trapped particles in the tumor and, most probably, further enhance the therapeutic effects. The retention efficiency could be further improved by using more sophisticated quadrupolar magnetic actuators, which could enhance the concentration in the whole tumor volume. The combination of magnetic concentration and enhanced photothermal effects endowed by the MAPSULES have enabled a complete eradication of the tumors using much lower drug and nanoparticle concentrations than the typical values used in the literature (between 100- and 500-fold lower than the therapeutic window of the free or PLGA encapsulated drug and between 13- and 3000-fold lower than previously described nanotherapies combining paclitaxel and light actuation). Note that although the employed power density of the 808 nm laser (*ca.* 1 W/cm^2^) is higher than the maximum recommended exposure values for humans at this wavelength, this power density is allowed for light irradiation in the second biological window at 1064 nm. In this wavelength, the MAPSULES exhibit an even higher photothermal efficiency, and penetration depth can be even higher for organs such as kidney, spleen, and liver.^48,49^ In regards to development perspectives toward clinical utilization, it is relevant to emphasize that the PLGA nanocarriers have already been approved by the FDA (e.g., Trelstar and Risperdal Consta). A next important step is the scaling up of the MAPSULES fabrication process. The self-assembly step takes less than 5 min and could be easily automatized to coat much larger surfaces and numbers of substrates. The fabrication process is currently limited by the Fe/SiO_2_ evaporation step, which takes *ca.* 25 min per wafer in a research-oriented e-beam evaporator. The duration of this process could be drastically reduced in industrial evaporators, which can load tens of larger wafers, and whose evaporation process can be continuously run without breaking the vacuum. Moreover, a combination of roll-to-roll self-assembly and material deposition, which is typically used in the tape recording or packaging industries, could be used for the cost-effective industrial production of the MAPSULES. Therefore, the promising therapeutic results of the MAPSULES enable envisaging externally controlled therapeutic pathways with minimal side effects, which could be easily extended to other drugs and tumors and even to other diseases.

## Methods

### Nanomaterial Preparation Section

#### Nanoprecipitation Synthesis Process of the Drug-Loaded PLGA Nanoparticles

First, a 5 mL organic solution containing 100 mg of carboxylic terminated PLGA (Resomer^R^ 502 H, Sigma), 2 mg of paclitaxel (Sigma), and 0.01% Tween80 (Sigma) in acetonitrile was added dropwise to a 10 mL aqueous solution containing 0.1% Solutol (Sigma) at room temperature. Following 30 min of stirring at 550 rpm, the acetonitrile was removed under reduced pressure using a rotary evaporator (Heidolph, Germany), leading to the formation of the paclitaxel-loaded PLGA nanoparticles. Paclitaxel-loaded PLGA nanoparticles were used without any further purification.

#### Fabrication of the MAPSULES

The fabrication approach was adapted from a previously developed method.^17,50^ Briefly, first, a 2 mg/mL suspension of drug-loaded PLGA nanoparticles in Milli-Q water was prepared and sonicated for 3 min. Meanwhile, the surfaces of 4″ silicon wafers (Siegert Wafer GmbH) were incubated in poly(diallyldimethylammonium chloride) (PDDA) 2% solution (Sigma) for 3 min. Then, the wafers were rinsed with water and blow dried with N_2_ gas, thereby yielding a monolayer of positively charged PDDA on the surface. Next, the PLGA nanoparticle dispersion was drop casted on the surface of the modified wafers and incubated for 3 min, followed by the same rinse and dry process with deionized water and N_2_ gas, to obtain a disordered and well separated self-assembled monolayer of nanoparticles on the surface. Any excess of material outside the loaded PLGA nanoparticles was totally removed during this rising and drying process.

Subsequently, electron beam evaporation (ATC-8E Orion, AJA International Inc.) was used to deposit 20 nm of Fe and 10 nm of SiO_2_ on the Si wafer coated with the self-assembled PLGA particles. Then, MAPSULES were dispersed from the wafer by mild ultrasonication in 2% poly(4-styrenesulfonic acid) solution and cleaned twice by using magnetic concentration with a FeNdB cylindrical magnet (diameter 12 mm, thickness 10 mm) to remove the supernatant, followed by redispersion in clean buffer.

### Morphological, Optical, Magnetic, and Colloidal Characterization

The morphology and distribution of the nanodomes on the Si wafer were studied through scanning electron microscopy (SEM) using a Quanta SEM 650 (Field Electron and Ion Company (FEI)) at 5 kV. Transmission electron microscopy, TEM, images were acquired in an FEI Tecnai F20 at 200 kV. The samples for the TEM analysis were prepared by placing a drop of the aqueous suspension of MAPSULES on a TEM grid and allowing it to air-dry for 2 h.

The zeta potential and the hydrodynamic size of the PLGA particles and the MAPSULES were obtained through dynamic light scattering using a Zetasizer Nano ZS (Malvern Instruments Ltd.).

The mass concentration of Fe in the nanodomes dispersions was determined by ICP-OES (Perking Elmer Optima 4300DV).

The visible-near-infrared (vis-NIR) spectroscopy studies of the nanodome dispersions were carried out using a Lambda25 spectrometer (PerkinElmer) in the 400 to 1100 nm range, covering the visible light and near-infrared regions.

The magnetic characterization of the nanodomes was performed on monolayers that were transferred from the Si wafer to a 10 mm polydimethylsiloxane (PDMS) substrate to eliminate the magnetic signal from the bilayer that was deposited on the wafer surface. Magnetization loops were acquired at room temperature using a vibrating sample magnetometer (MicroSense, LOT QuantumDesign) with a maximum applied field of 20 kOe. The measurements were performed by applying the field either parallel or perpendicular to the sample, i.e., in-plane or out-of-plane conditions.

### Magnetic Trapping in the Microfluidic Channel

The magnetic trapping experiments were performed by setting a spherical 19 mm diameter FeNdB permanent magnet (7.1 kOe at the surface and field gradient according to Figure S2C) attached to a narrow microfluidic channel (3 mm wide, 0.8 mm height). A MAPSULES dispersion (total volume of 1 mL) was controllably flowed by a syringe pump through the microfluidic channel at different flow rates. The experimental setup is shown in Figure S2. The optical absorbance spectra of the sample before and after the trapping experiments were measured to establish the concentration of nanoparticles in the dispersion and the trapping efficiency.

### Nuclear Magnetic Resonance

^1^H-magnetic resonance imaging (MRI) studies were performed in a 70 kOe Bruker BioSpec 70/30 USR (Bruker BioSpin GmbH, Ettlingen, Germany) system equipped with a mini-imaging gradient set (4000 Oe/m) using a linear volume coil with a 72 mm inner diameter. The magnetic resonance data were acquired and processed on a Linux computer using Paravision 5.1 software (Bruker BioSpin GmbH, Ettlingen, Germany). For the relaxivity measurements of the MAPSULES, phantoms containing various nanodome concentrations in 1% agar and PBS (1× ) mixture were prepared. The magnetic resonance images were obtained from two 2.5 mm slice thickness coronal sections with a field of view (FOV) of 90 × 60 mm^2^. Only transverse relaxation time (*T*_2_) measurements have been performed. A multislice multiecho sequence was used, with repetition time (TR) = 3 s, time of echo (TE) values between 10 and 600 ms in steps of 10 ms, and MTX = 128 × 128. Data were fitted to exponential curves to obtain the *T*_2_ relaxation times. Transverse relaxivity values, *r*_2_, were obtained as the slope of the linear regression of the relaxation rates (*r*), as the inverse of the relaxation times (*r*_2_ = 1/*T*_2_) versus Fe concentration.

A similar approach was used to image *T*_2_ contrast in nuclear magnetic resonance signal of the MAPSULES *in vivo* in mice. MRI experiments were performed at the Wohl Institute for Translational Medicine at Hadassah Hebrew University Medical Center. Images were acquired on a 7 T 240 mm bore, cryogen-free MR scanner based on the proprietary dry magnet technology (MR Solutions, Guildford, UK) using a 38 mm inner diameter, 70 mm length mouse whole body volume coil. In detail, the unloaded MAPSULES nanocapsules were injected via the tail vein with a total volume of 150 μL with a total mass concentration of 2.5 mg/kg. For MRI acquisition, mice were anesthetized with isoflurane vaporized with O_2_. Isoflurane is used at 3% for induction and at 1–2% for maintenance. The mice were positioned on a heated bed, which allowed for continuous anesthesia and breathing rate monitoring throughout the entire scan period. Coronal *T*_1_-weighted (T_1_W) spin–echo images were acquired for anatomical segmentation purposes (RT/ET = 1100/11 ms, FOV = 60 mm, in-plane resolution = 230 μm, slice thickness = 1 mm). Coronal and axial T_2_*W gradient echo images were acquired for MAPSULES detection (RT/ET = 300/10 ms, FOV = 60/35 mm, slice thickness = 1 mm). The MRI tests were conducted at different times before and after (2, 24, 48, and 72 h) the MAPSULES injection.

### Photothermal Heating Efficiency Measurement

A custom-made photothermal testing system was used to determine the photothermal conversion efficiency of nanocapsules in water as previously described.^16^ The system consisted of (i) a NIR laser diode with the selected emission wavelength (808 nm, L808P500MM, Thorlabs, or 1064 nm, KA64FAMFA, Thorlabs) driven by a laser diode controller (ITC4005, Thorlabs), (ii) an optical collimating and aligning system, (iii) an infrared camera (ETS320, FLIR) to monitor the temperature variations at the liquid surface, (iv) a power meter (PM100D, Thorlabs), and (v) a computer with the data acquisition software. The laser incident power upon the samples was set to 200 mW for both lasers, and the temperature of the solution and the power transmitted through the sample were continuously monitored during the assays. The photothermal conversion efficiency η was calculated as follows:^51^
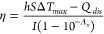
1where *h* is the heat transfer coefficient, *S* is the laser irradiating surface, Δ*T*_*max*_ is the maximum temperature change optically induced, *Q*_*dis*_ is the heat dissipation due to the experimental setup, *I* is the laser incident power (200 mW), and *A*_*x*_ is the absorbance at the irradiated wavelength (*x* = 808 or 1064 nm).

In order to get the value of η, *hS* for heat dissipating should be calculated. During the cooling process, since there is no light irradiating the nanoparticles, the dispersion will reach *T_amb_*:
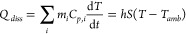
2

Here we can define the time constant τ_*s*_ and the dimensionless parameter θ to simplify the integral as follows:
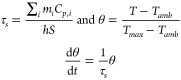
3

Setting the boundary conditions for the cooling process, *t* = 0, θ = 1, the equation can be solved as eq 4, from which a linear relationship can be deduced to obtain the τ_*s*_ value; consequently, *hS* can be calculated and introduced in eq 1 to calculate the photothermal efficiency.
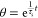
4

The total volume of the MAPSULES dispersion was 600 μL. The measured extinction values of the MAPSULES dispersion at 808 and 1064 nm were 0.2192 and 0.1837 (Figure S4A), respectively. The constant time of the system was determined as 280 s, as shown in Figure S4B.

### Cell Culture

MDA-MB-231 breast adenocarcinoma tumor cells were purchased from ATCC (VA, USA) and maintained in DMEM, supplemented with 10% fetal calf serum (FCS) (Gipco, Brazil) media with 1% penicillin/streptomycin (Biological industries, Israel). For comparison of viability of the unloaded and paclitaxel-loaded PLGA nanoparticles and MAPSULES, 2 × 10^4^ MDA-MB-231 cells were plated in a 96 well plate and incubated for 24 h. The PLGA and MAPSULES treatments were introduced at identical particle concentrations with 10 μg/mL of PLGA nanoparticles and 0.08 μg/mL of paclitaxel, and the plates were incubated for an additional 48 h before the viability was evaluated using a MTT assay. The MTT (3-(4,5-dimethylthiazol-2-yl)-2,5-diphenyl- tetrazolium bromide) was added to the cell cultures (final concentration 0.5 mg/mL) and incubated for another 4 h. Then, the supernatant was removed, and DMSO was added allowing the crystals to dissolve completely for absorbance read at 570 nm by a SPECTRAFluor Plus plate reader (Tecan, San Jose, CA, USA). Cells were also visualized using an inverted microscope (Nikon Eclipse Ti2, Japan) 4 h after incubation with the MAPSULES. In order to measure the effects of the magnetic concentration and photothermal actuation of the MAPSULES on the cells viability, 5 × 10^4^ cells were plated in a 35 mm Petri dish and incubated for 24 h before introducing magnets under the center of the well (12.7 mm spherical FeNdB magnet, 30 min) and/or laser radiation (wavelength 808 nm, beam diameter 11 mm, power density 0.95 W/cm^2^, 10 min). The temperature was monitored by an infrared thermal camera (8320P, Infrared Cameras Inc. (ICI), Beaumont, TX, USA). The cells were incubated for 4 h following the treatments and then washed and incubated for another 24 h before the viability was evaluated. Cells were stained by calcein AM (2 μmol/L, 30 min), washed with PBS, and the fluorescent signal was measured by aerial scan using a plate reader (ex/em 488 nm/520 nm). The cell viability was also evaluated by MTT (as mentioned).

### *In Vivo* Experiment

For the *in vivo* analysis of the therapeutic effect, 8-week-old Hsd:Athymic Nude-Foxn1^nu^ female mice were purchased (Harlan, Rehovot, Israel), acclimated for a week, and stored in a specific pathogen-free (SPF) environment. All animal procedures were carried out in accordance with the institutional and national guidelines, and protocols were approved by the ethics committee of the Hebrew University Ein Kerem Medical School IACUC (protocol MD-21-16572-5). Nude mice were inoculated subcutaneously with 4 × 10^6^ MDA-MB-231 cells, and the tumor volumes were monitored and measured according to the equation:
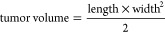


When tumor volumes reached approximately 100 mm^3^, mice were randomly divided into groups and subjected to 150 μL tail intravenous injection of six different treatments: (i) PBS (PBS), (ii) PBS plus laser (PBS+L), (iii) MAPSULES (MPs), (iv) MAPSULES plus laser (MPs+L), (v) MAPSULES plus magnet (MPs+M), and (vi) MAPSULES plus magnet and laser (MPs+M+L). The magnetic actuated groups, MPs+M and MPs+M+L, were exposed to an N45 nickel-plated FeNdB cylindrical magnet (diameter 12 mm, thickness 10 mm) placed next to the tumor for 2 h, while the mice were anesthetized on a warming pillow set to 33 °C. The laser actuated groups were radiated for 20 min by an 808 nm continuous wave laser beam (0.95 W/cm^2^, beam diameter 11 mm) and the temperature was monitored using a thermal camera. The tumors were measured three times a week for 21 days or until the exclusion criterion of 1000 mm^3^ was reached (end points). At the end points, mice were sacrificed, imaged and the tumors were removed, photographed and weighted.

## Supporting Information Available

MAPSULES characterization, trapping analysis, degradation, photothermal efficiency calculation, mice weight evolution, and tumor progression (PDF)
