# Supplementary material for: Efficient Tumor Eradication at Ultralow Drug Concentration via Externally Controlled and Boosted Metallic Iron Magnetoplasmonic Nanocapsules
Source: ACS Nano. 2022 Dec 5;17(3):1946–58. doi: 10.1021/acsnano.2c05733 (PMC9933591; doi:10.1021/acsnano.2c05733)
Supplement: Supplementary file 1 — nn2c05733_si_001.pdf [file nn2c05733_si_001.pdf]

Supporting information for

Efficient tumor eradication at ultralow drug concentration via externally controlled and boosted metallic iron magneto-plasmonic nanocapsules

---

Arnon Fluksman[1‡], Aritz Lafuente[2,3‡], Zhi Li[2†], Jordi Sort[3,4], Silvia Lope-Piedrafita[3], Maria José Esplandiu[2], Josep Nogues[2,4], Alejandro G. Roca[2], Ofra Benny[1*], Borja Sepulveda[5*]

‡ Equal contribution

[1] Institute for Drug Research (IDR), School of Pharmacy, Faculty of Medicine, The Hebrew University of Jerusalem, 9190501, Jerusalem, Israel.

[2] Catalan Institute of Nanoscience and Nanotechnology (ICN2), CSIC and BIST, Campus UAB, 08193, Bellaterra, Barcelona, Spain.

[3] Universitat Autònoma de Barcelona, Campus UAB, 08193, Cerdanyola del Vallès, Barcelona, Spain.

[4] ICREA, Pg. Lluís Companys 23, 08010, Barcelona, Spain

[5] Instituto de Microelectronica de Barcelona (IMB-CNM, CSIC), Campus UAB, 08193, Bellaterra, Barcelona, Spain

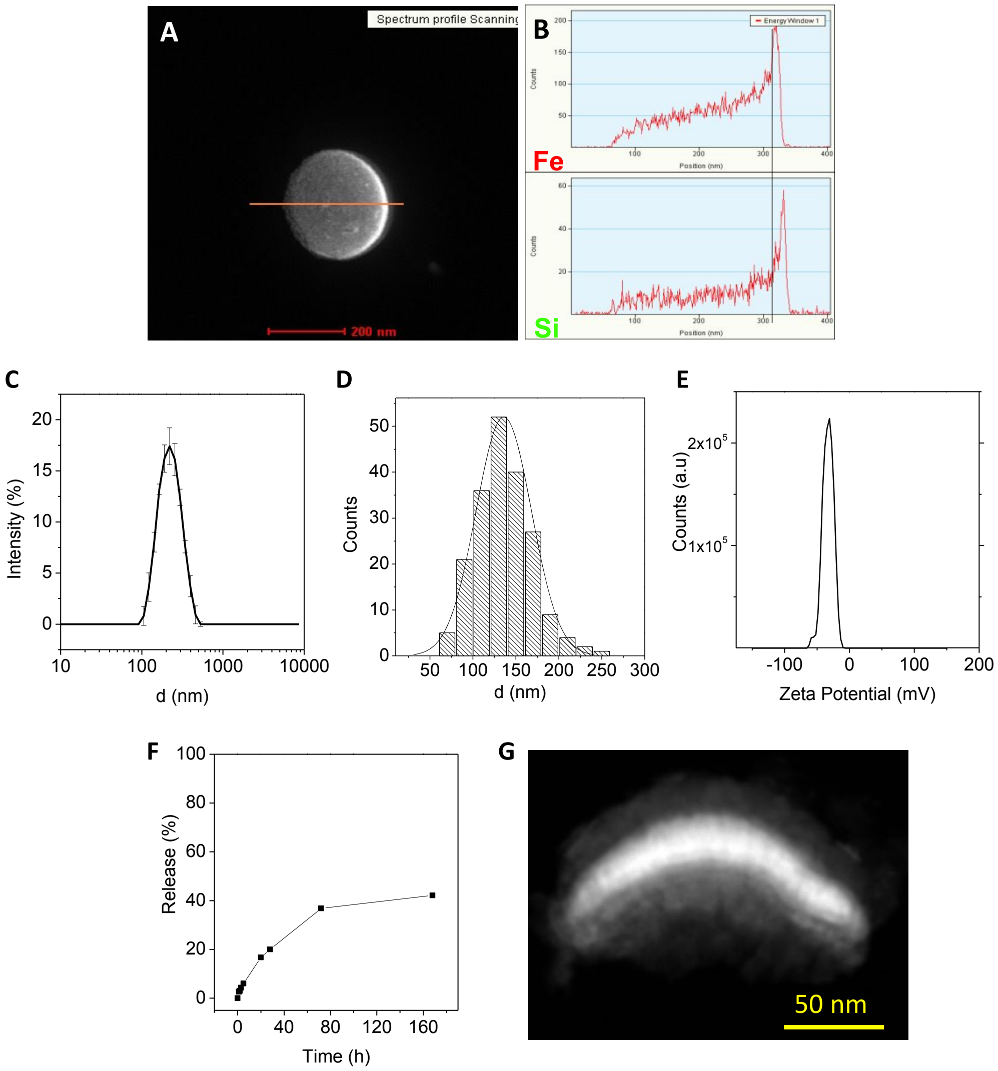

Figure S1. MAPSULES structural and colloidal characterization and drug release. A) TEM image of a MAPSULE for the EDX spectrum profile scanning analysis to determine the thickness of the Fe and SiO_2 layers, and B) profiles centered at the Fe and Si energies, showing thickness of 22 nm and 10 nm for the Fe and SiO_2 layers, respectively. C) Nanoparticles size distribution by DLS and D) by SEM. E) Zeta potential distribution. F) Paclitaxel release from the PLGA nanoparticles during the first 7 days. G) TEM picture showing the degradation of the MAPSULES at room temperature in deionized water after 30 days.

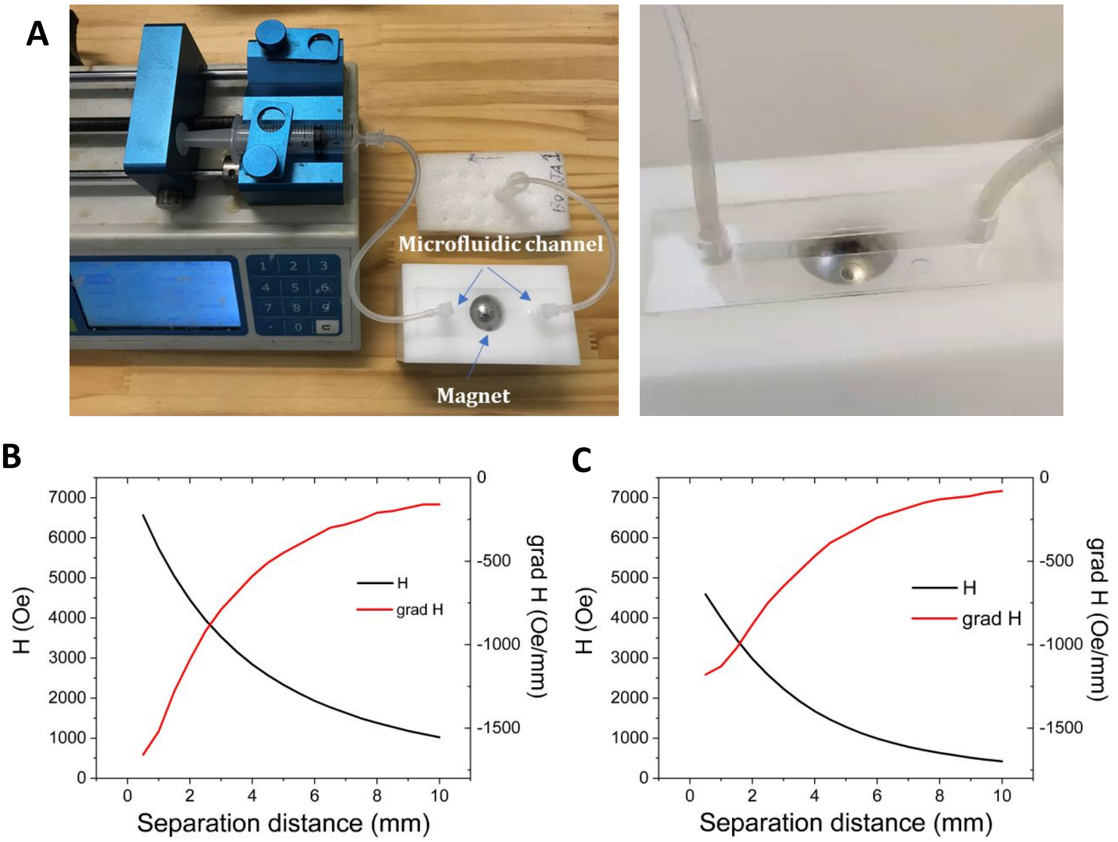

Figure S2. Magnetic trapping experimental conditions. (A) Experimental setup for magnetic trapping experiments in magnetic fluidic channel (3 mm wide, 0.8 mm height). (B) and (C) Magnetic field distribution and magnetic field gradient as a function of the separation distance for the spherical and cylindrical magnets employed in the particle trapping experiment in microfluidic channels and in the *in vivo* experiments, respectively.

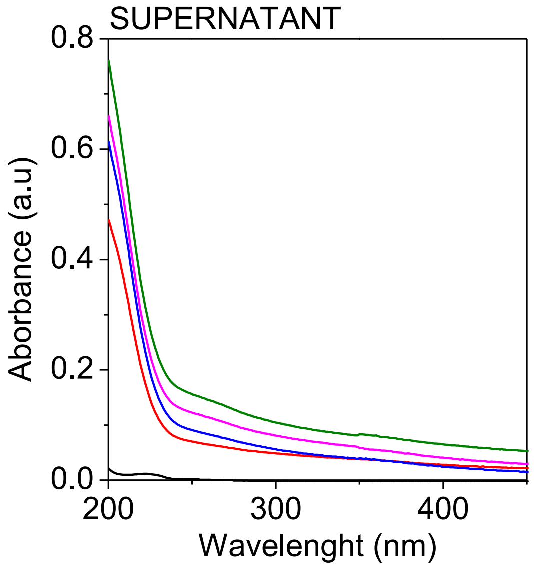

Figure S3. MAPSULES degradation evolution. Evolution of the MAPSULES and supernatant absorbance as a function of time at 37°C.

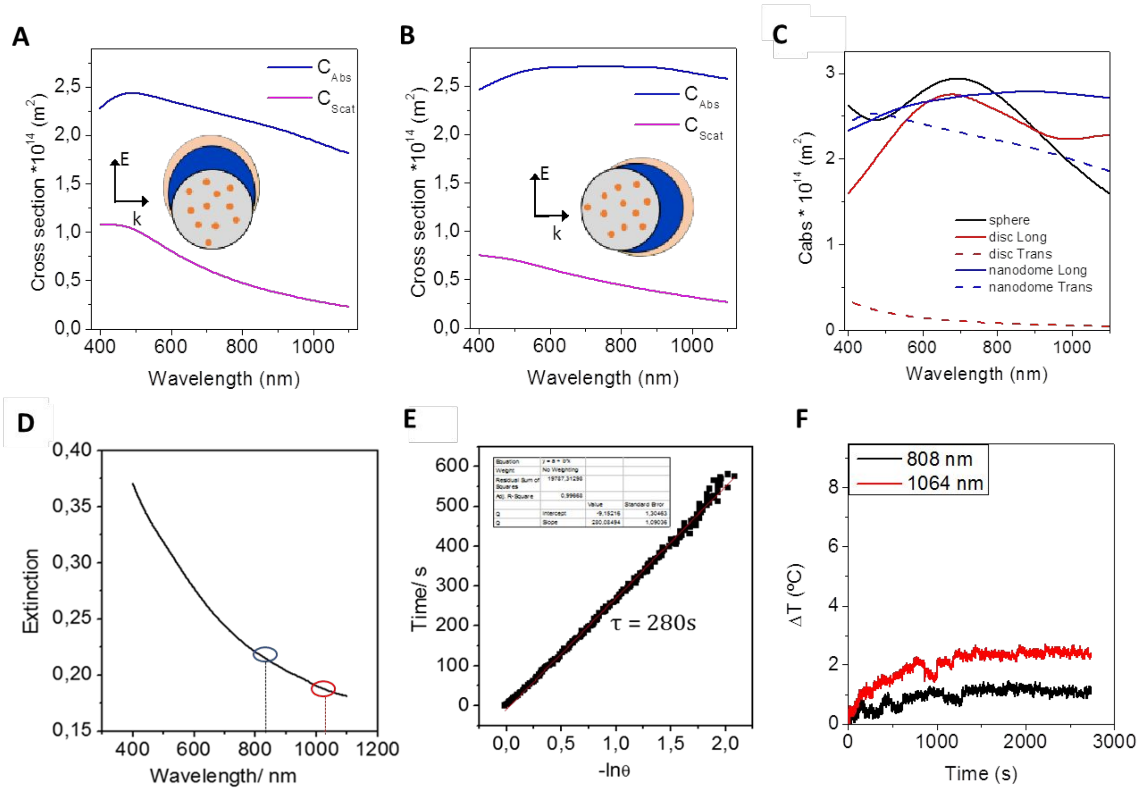

Figure S4. Theoretical analysis of the absorption and scattering cross-sections and parameters for the calculation of the photothermal efficiency. (A) and (B) Theoretical simulations through finite domain time domain (FDTD) of scattering (C_{scat}) and Absorption (C_{abs}) cross sections of the MAPSULES for different orientations of the nanocapsules with respected to the polarization of the incident light. (C) FDTD simulations comparing the absorption cross section for iron nanospheres (diameter 150 nm), nanodiscs (diameter 150 nm, height 20 nm) and MAPSULES. (D) Experimental extinction curve of the MAPSULES dispersion in water for measuring the optical heating efficiencies under 808 nm and 1064 nm laser illumination. (E) The time constant for heat transfer of the system is determined to be τ = 280s. (F) Optical heating of water in the first and second biological windows with 808 nm and 1064 nm lasers at a fixed power of 200 mW. (0.95 W/cm^2).

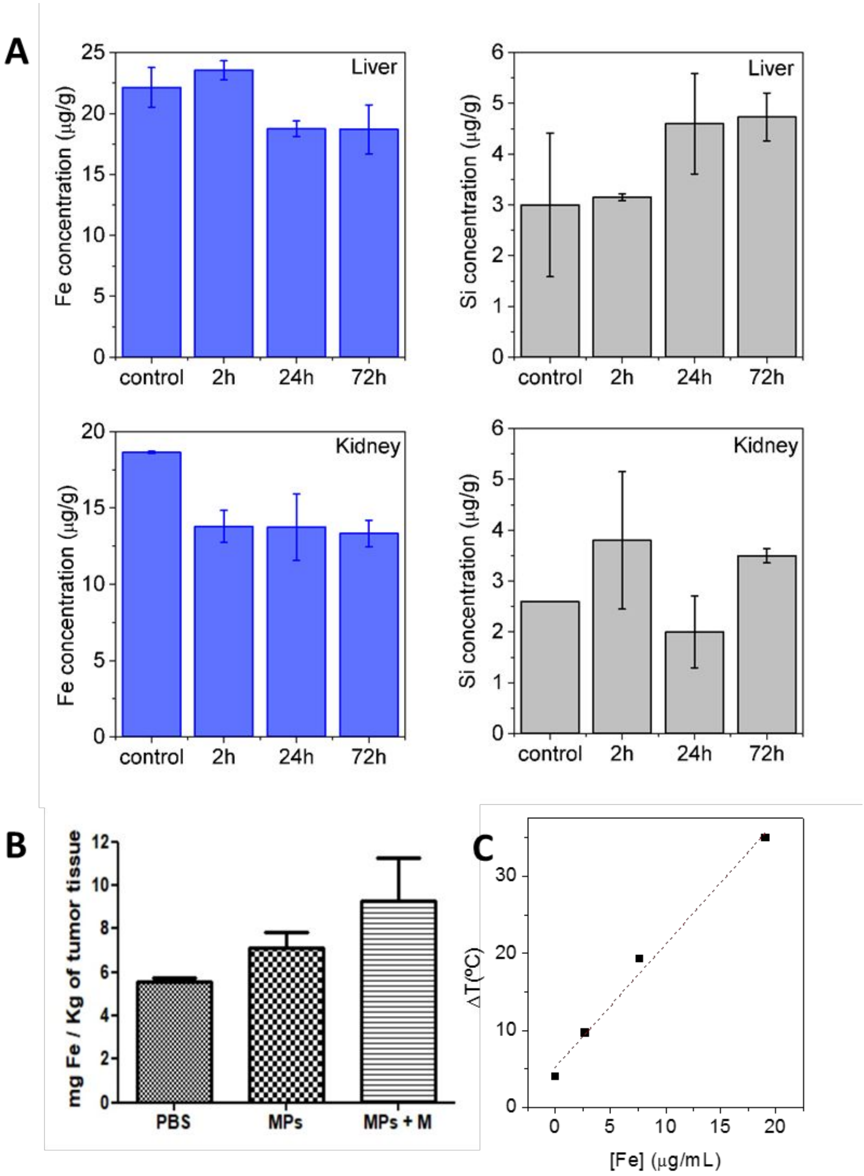

Figure S5. *In vivo* biodistribution analysis by mass spectrometry. (A) Analysis of the iron and silicon content in the liver and kidneys before injection (control) and at different time points post injection (2h, 24h and 72h). (B) Comparison of the iron content in the tumor and in the PBS control with respect to the passive injection and the magnetic accumulation conditions. The tumors were harvested 24h after the injection. (C) Photoinduced temperature increase in a MAPSULES dispersion as a function of the particle concentration expressed in iron mass per volume, induced by the 808 nm laser irradiation with 0.95 W power in a volume of 650 µL for 20 min.

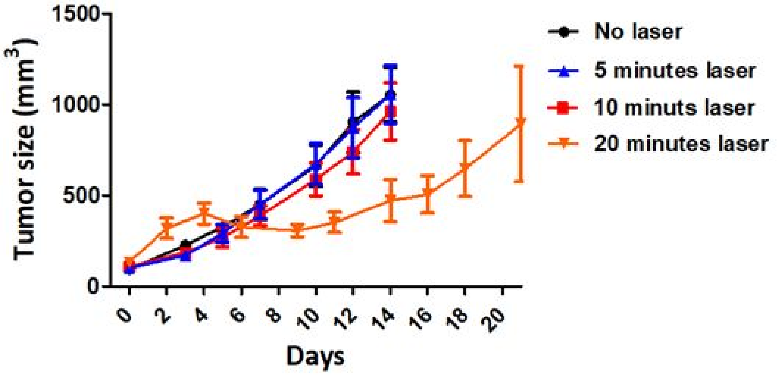

Figure S6. In vivo analysis of the photothermal effects without injected MAPSULES. Comparative analysis of the tumor growth after illumination of the tumor for 5, 10 and 20 minutes (power density 0.95 W/cm^2) with respect to the non-illuminated control.

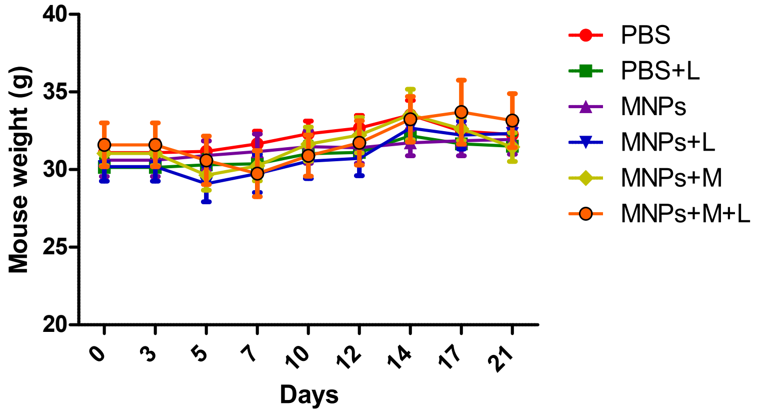

Figure S7. In vivo safety analysis. Body weight of the MDA-MB-231 tumor bearing mice following the different treatments.

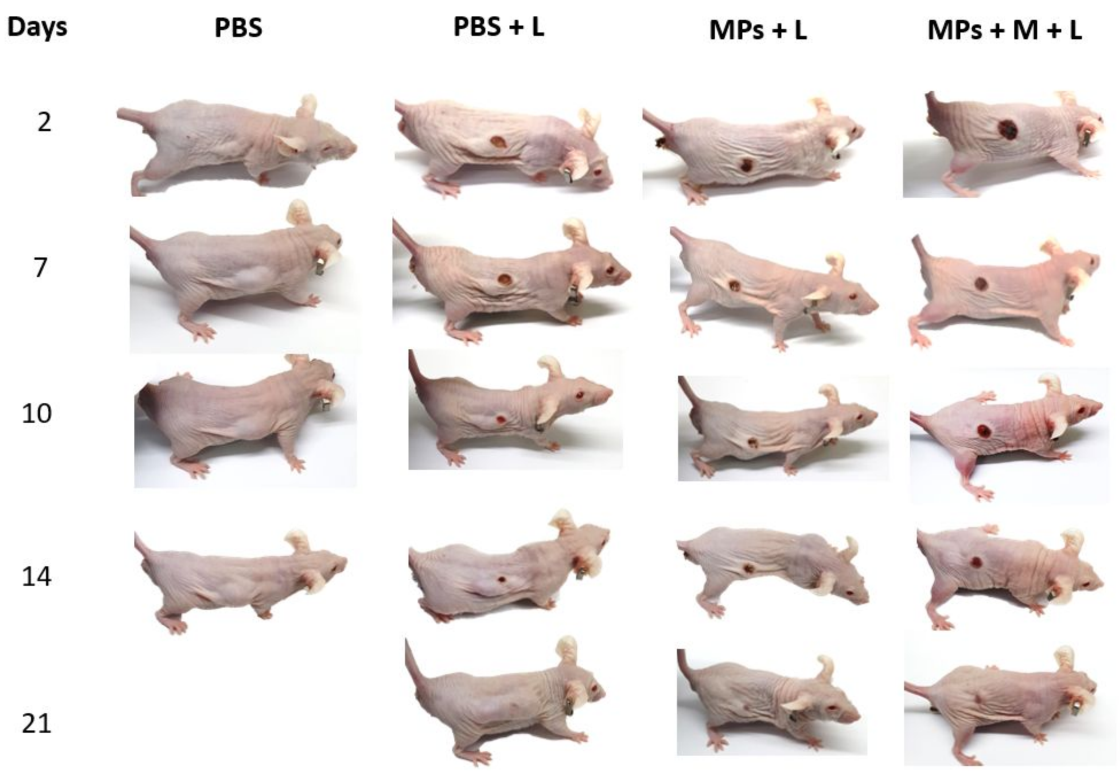

Figure S8. Tumor progression. Images of the light treated mice over time showing tumor growth and skin burn recovery following the different treatments.
